# High-quality permanent draft genome sequence of *Ensifer* sp. PC2, isolated from a nitrogen-fixing root nodule of the legume tree (Khejri) native to the Thar Desert of India

**DOI:** 10.1186/s40793-016-0157-7

**Published:** 2016-06-23

**Authors:** Hukam Singh Gehlot, Julie Ardley, Nisha Tak, Rui Tian, Neetu Poonar, Raju R. Meghwal, Sonam Rathi, Ravi Tiwari, Wan Adnawani, Rekha Seshadri, T. B. K. Reddy, Amrita Pati, Tanja Woyke, Manoj Pillay, Victor Markowitz, Mohammed N. Baeshen, Ahmed M. Al-Hejin, Natalia Ivanova, Nikos Kyrpides, Wayne Reeve

**Affiliations:** BNF and Stress Biology Lab., Department of Botany, J.N. Vyas University, Jodhpur, 342001 India; Centre for Studies, Murdoch University, Murdoch, Western Australia Australia; DOE Joint Genome Institute, Walnut Creek, California USA; Biological Data Management and Technology Center, Lawrence Berkeley National Laboratory, Berkeley, California USA; Department of Biological Sciences, Faculty of Science, King Abdulaziz University, Jeddah, Saudi Arabia

**Keywords:** Root-nodule bacteria, Nitrogen fixation, Symbiosis, *Ensifer*, *Prosopis*

## Abstract

*Ensifer* sp. PC2 is an aerobic, motile, Gram-negative, non-spore-forming rod that was isolated from a nitrogen-fixing nodule of the tree legume *P. cineraria* (L.) Druce (Khejri), which is a keystone species that grows in arid and semi-arid regions of the Indian Thar desert. Strain PC2 exists as a dominant saprophyte in alkaline soils of Western Rajasthan. It is fast growing, well-adapted to arid conditions and is able to form an effective symbiosis with several annual crop legumes as well as species of mimosoid trees and shrubs. Here we describe the features of *Ensifer* sp. PC2, together with genome sequence information and its annotation. The 8,458,965 bp high-quality permanent draft genome is arranged into 171 scaffolds of 171 contigs containing 8,344 protein-coding genes and 139 RNA-only encoding genes, and is one of the rhizobial genomes sequenced as part of the DOE Joint Genome Institute 2010 *Genomic Encyclopedia for Bacteria and Archaea-Root Nodule Bacteria* (GEBA-RNB) project proposal.

**Electronic supplementary material:**

The online version of this article (doi:10.1186/s40793-016-0157-7) contains supplementary material, which is available to authorized users.

## Introduction

The genus *Prosopis* (family *Leguminosae*, sub-family *Mimosoideae* [1]) comprises about 44 species that are widely distributed in the world’s semi-arid regions, mostly in North and South America with a few species found in Africa and south west Asia [2–4]. Several species have been widely introduced throughout the world over the last 200 years [5]. *Prosopis* may have evolved from *P. africana* (Guill. & Perr.) Taub., in which various character traits and small genome size (392–490 Mbp) indicate that it is a primitive species [2]. According to Burkart [2], *Prosopis* is an old genus that diverged early into several principal lineages, with some of these lineages producing more recent episodes of speciation. This is supported by a recent molecular dating analysis that places the divergence of the New World *Prosopis* Sections during the Oligocene (33.9 to 23.03 Mya) [6], which is remarkably ancient considering that the subfamily *Mimosoideae* originated between 42–50 Mya [7]. Section Prosopis consists of three species, *Prosopis cineraria* (L.) Druce*, P. farcta* (Banks et Sol.) Eig. and *P. koelziana* Burkart, which are native to North Africa and Asia [6].

*P. cineraria* is endemic to arid and semi-arid regions of the Indian Thar Desert and is designated as the state tree of Rajasthan [8]. It symbolizes the sacred mythological “Kalpa Vriksh” (wish tree) of the desert and is historically important, as it has been worshiped since ancient times by many rural communities in these arid regions. *P. cineraria* is a multipurpose tree used as food, fodder, shelter and medicine by the local inhabitants. It is an important component of agro forestry, agrisilvicultural and silvopastoral systems in the alkaline soil of the Thar Desert. The tree is extremely drought and salt tolerant, having a deep root system (>100 metres) that helps in acquiring nutrients and moisture from deeper soil layers. It produces green pods that are rich in nutrients and antioxidants and eaten as a vegetable in the hot summer [9]. *P. cineraria* is a good candidate for rehabilitation of dry, marginal or degraded lands of low fertility and/or high salinity. It plays a vital role as a soil binder in the stabilization of sand dunes and enriches poor desert soil by fixing atmospheric nitrogen in association with its rhizobial microsymbionts [10–13].

*Prosopis* is a promiscuous genus, being nodulated by a wide range of taxonomically diverse rhizobia. Mesquite (Torr.) in the Sonoran Desert, California is nodulated by diverse strains of fast- and slow-growing rhizobia [14]. *Mesorhizobium chacoense*CECT 5336^T^ is a microsymbiont of *Prosopis alba* Griseb. growing in the Chaco Arido region in Argentina [15], whereas in Spain is nodulated by strains of *Ensifer medicae**,**E. meliloti* and *Rhizobium giardinii* [16]. In Africa, the introduced *Prosopis* species *P. chilensis* (Molina) Stuntz*, P. cineraria, P. juliflora* (Sw.) DC. and *P. pallida* (Willd.) Kunth are reported to nodulate with strains of *Ensifer arboris*, *E. kostiense,**E. saheli* and *E. terangae* [17, 18] and *P. juliflora* also forms effective symbioses with strains of *Mesorhizobium plurifarium* [19] and *Rhizobium etli* [20]. Nodulation of *P. cineraria* growing in its native range was first described by Basak and Goyal [10]. Recently, *P. cineraria* and other native legumes growing in the alkaline soils of the Thar desert have been reported to nodulate with a dominant novel group of *Ensifer* strains (PC2, TW10, TP13, RA9, TV3 and TF7) that are closely related to African and Australian *Ensifer* strains on the basis of 16S rRNA sequence similarity, but form a distinct, well-separated cluster [21, 22].

The indigenous rhizobia of wild tree legumes growing in such arid and harsh environments have superior tolerance to abiotic factors such as salt stress, elevated temperatures and drought and can be used as inoculants for wild as well as crop legumes cultivated in reclaimed desert lands [10]. Because of its ability to nodulate the keystone species *P. cineraria* as well as crop legumes such as *Vigna radiata* (L.) R.Wilczek and *V. unguiculata* (L.) Walp. [21], strain PC2 has therefore been selected as part of the DOE Joint Genome Institute 2010 *Genomic Encyclopedia for**Bacteria**and Archaea-Root Nodule Bacteria* (GEBA-RNB) sequencing project [23]. Here we present a summary classification and a set of general features for *Ensifer* sp. strain PC2, together with a description of its genome sequence and annotation.

## Organism information

### Classification and features

**Fig. 1:**
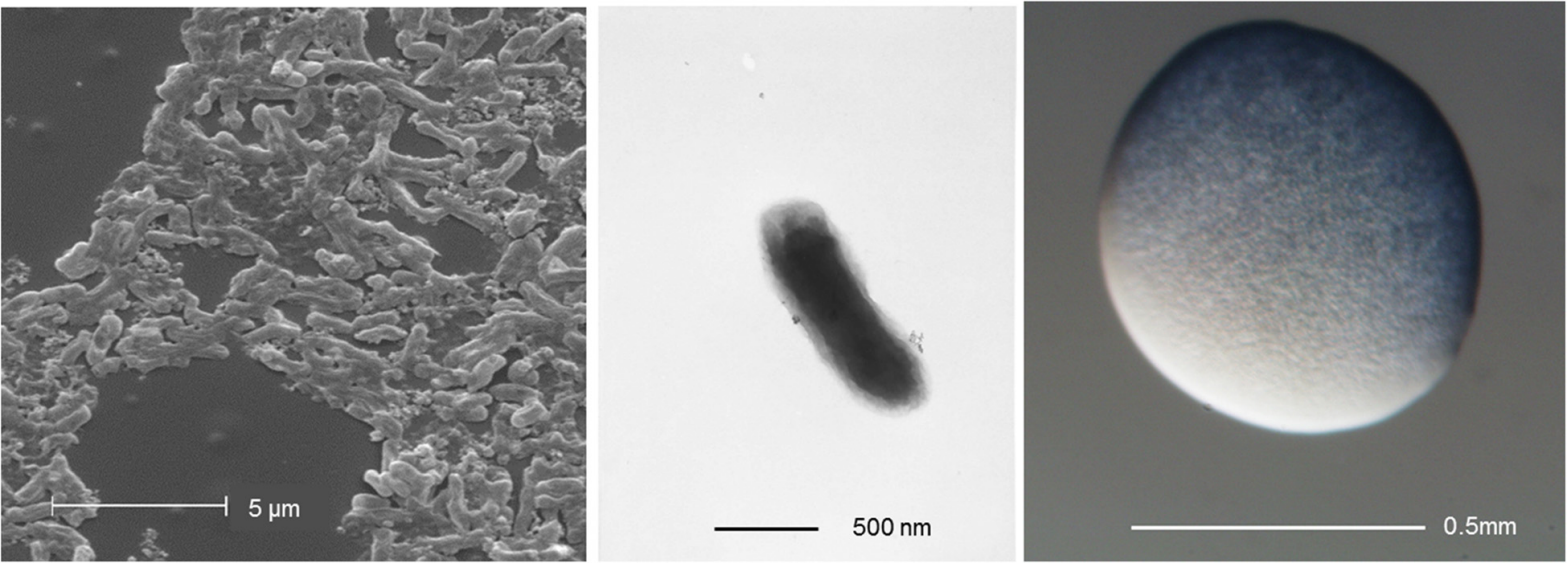
Images of *Ensifer* sp. PC2 using scanning (Left) and transmission (Center) electron microscopy and the appearance of colony morphology on solid media (Right)

*Ensifer* sp. PC2 is a motile, Gram-negative strain in the order *Rhizobiales* of the class *Alphaproteobacteria*. The rod shaped form (Fig. 1 Left and Center) has dimensions of approximately 0.3-0.5 μm in width and 1.25-1.5 μm in length. It is fast growing, forming colonies within 3–4 days when grown on half strength Lupin Agar [24], tryptone-yeast extract agar (TY) [25] or a modified yeast-mannitol agar (YMA) [26] at 28 °C. Colonies on ½LA are white, opaque, slightly domed and slightly mucoid with smooth margins (Fig. 1 Right).

**Fig. 2:**
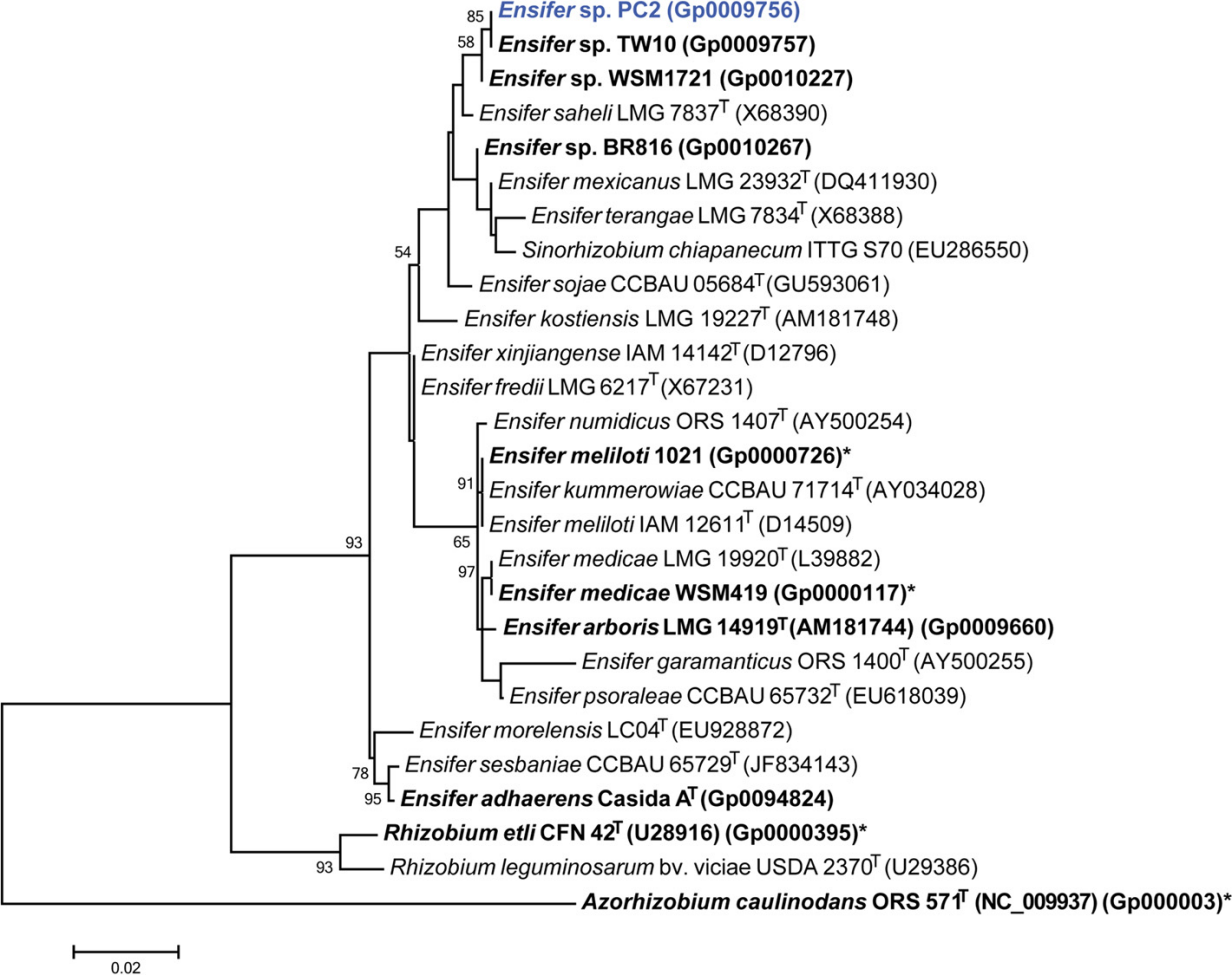
Phylogenetic tree showing the relationship of *Ensifer* sp. PC2 (shown in bold blue print) to *Ensifer* spp. and other root nodule bacteria species in the order *Rhizobiales*, based on aligned sequences of the 16S rRNA gene (1,283 bp internal region). (The species name “*Sinorhizobium chiapanecum*” has not been validly published.) *Azorhizobium caulinodans* ORS 571^T^ was used as an outgroup. All sites were informative and there were no gap-containing sites. Phylogenetic analyses were performed using MEGA, version 6 [44]. The tree was built using the Maximum-Likelihood method with the General Time Reversible model [45]. Bootstrap analysis [46] with 500 replicates was performed to assess the support of the clusters. Type strains are indicated with a superscript T. Strains with a genome sequencing project registered in GOLD [29] are in bold font and the GOLD ID is provided after the GenBank accession number, where this is available. Finished genomes are indicated with an asterisk.

Figure 2 shows the phylogenetic relationship of *Ensifer* sp. PC2 in a 16S rRNA sequence based tree. This strain is the most similar to *Ensifer saheli*LMG 7837^T^ based on the 16S rRNA gene alignment, with sequence identities of 99.41 % over 1,366 bp, as determined using the EzTaxon-e database, which contains the sequences of validly published type strains [27]. The PC2 16S rRNA gene sequence has 100 % sequence identity with that of another Indian Thar Desert rhizobial strain, *Ensifer* sp. TW10, isolated from a nodule of the perennial legume *Tephrosia wallichii* [22]. Minimum Information about the Genome Sequence for PC2 is provided in Table 1 and Additional file 1: Table S1.

**Table 1:** Classification and general features of *Ensifer* sp. PC2 in accordance with the MIGS recommendations [47] published by the Genome Standards Consortium [48]

MIGS ID	Property	Term	Evidence code^a^
	Current classification	Domain Bacteria	TAS [49]
		Phylum *Proteobacteria*	TAS [50, 51]
		Class *Alphaproteobacteria*	TAS [52, 53]
		Order *Rhizobiales*	TAS [54]
		Family *Rhizobiaceae*	TAS [55]
		Genus *Ensifer*	TAS [56–58]
		Species *Ensifer* sp.	IDA
		Strain: PC2	TAS [21]
	Gram stain	Negative	IDA
	Cell shape	Rod	IDA
	Motility	Motile	IDA
	Sporulation	Non-sporulating	NAS
	Temperature range	10-40 °C	IDA
	Optimum temperature	28 °C	IDA
	pH range; Optimum	5-9.5; 6.5-8	IDA
	Carbon source	Mannitol, tryptone, yeast extract	TAS [21]
MIGS-6	Habitat	Soil; root nodule on host (*Prosopis* (L.) Druce)	TAS [21]
MIGS-6.3	Salinity	0.89-2.0 % (w/v)	NAS
MIGS-22	Oxygen requirement	Aerobic	TAS [21]
MIGS-15	Biotic relationship	Free living, symbiotic	TAS [21]
MIGS-14	Pathogenicity	Biosafety level 1	TAS [59]
MIGS-4	Geographic location	Jodhpur, Indian Thar Desert	TAS [21]
MIGS-5	Sample collection	October, 2009	TAS [21]
MIGS-4.1	Latitude	26.27061	TAS [21]
MIGS-4.2	Longitude	73.021177	TAS [21]
MIGS-4.3	Depth	0-10 cm	NAS
MIGS-4.4	Altitude	234 m	TAS [21]

^a^Evidence codes – IDA: Inferred from Direct Assay; TAS: Traceable Author Statement (i.e., a direct report exists in the literature); NAS: Non-traceable Author Statement (i.e., not directly observed for the living, isolated sample, but based on a generally accepted property for the species, or anecdotal evidence). These evidence codes are from the Gene Ontology project [60], [http://geneontology.org/page/guide-go-evidence-codes]

#### Symbiotaxonomy

*Ensifer* sp. strain PC2 is able to nodulate and fix nitrogen with both mimosoid and papilionoid legume hosts. It is interesting to note that sp. PC2 is able to nodulate and fix nitrogen with *Acacia saligna* (Labill.) Wendl., a promiscuous legume tree that mainly nodulates with species of in its native southwestern Australia range [28]. PC2 also effectively nodulates the Central American mimosoid tree *Leucaena leucocephala* (Lam.) de Wit. PC2 appears to be a relatively promiscuous strain that has potential to be used as an inoculant for crop legumes species such as *Vigna radiata* (L.) Wilczek and *V. unguiculata* (L.) Walp.. The symbiotic characteristics of sp. strain PC2 on a range of selected hosts are summarised in Additional file 2: Table S2.

## Genome sequencing information

### Genome project history

This organism was selected for sequencing on the basis of its environmental and agricultural relevance to issues in global carbon cycling, alternative energy production, and biogeochemical importance, and is part of the *Genomic Encyclopedia of**Bacteria**and Archaea, The Root Nodulating**Bacteria* chapter project at the U.S. Department of Energy, Joint Genome Institute. The genome project is deposited in the Genomes OnLine Database [29] and a high-quality permanent draft genome sequence is deposited in IMG [30]. Sequencing, finishing and annotation were performed by the JGI [31]. A summary of the project information is shown in Table 2.

**Table 2:** Genome sequencing project information for *Ensifer* sp. PC2

MIGS ID	Property	Term
MIGS-31	Finishing quality	High-quality draft
MIGS-28	Libraries used	Pacbio SMRTbell^TM^ library
MIGS-29	Sequencing platforms	Pacific Biosciences RS
MIGS-31.2	Fold coverage	181.5x
MIGS-30	Assemblers	HGAP (version: 2.0.12.0.1)
MIGS-32	Gene calling methods	Prodigal 1.4
	Locus Tag	B077 [http://www.ncbi.nlm.nih.gov/bioproject/?term=B077]
	GenBank ID	LATE00000000
	GenBank Date of Release	Apr 20 2015
	GOLD ID	Gp0009756 [https://gold.jgi-psf.org/project?id=9756]
	BIOPROJECT ID	PRNJA169749
MIGS-13	Source Material Identifier	PC2, WSM4384
	Project relevance	Symbiotic N_2_ fixation, agriculture

### Growth conditions and genomic DNA preparation

*Ensifer* sp. PC2 was streaked onto TY solid medium [25, 32] and grown at 28 °C for three days to obtain well grown, well separated colonies, then a single colony was selected and used to inoculate 5 ml TY broth medium. The culture was grown for 48 h on a gyratory shaker (200 rpm) at 28 °C. Subsequently 1 ml was used to inoculate 60 ml TY broth medium and grown on a gyratory shaker (200 rpm) at 28 °C until OD_600nm_ 0.6 was reached. DNA was isolated from 60 ml of cells using a CTAB bacterial genomic DNA isolation method [http://jgi.doe.gov/collaborate-with-jgi/pmo-overview/protocols-sample-preparation-information/]. Final concentration of the DNA was 0.5 mg ml^−1^.

### Genome sequencing and assembly

The draft genome of sp. PC2 was generated at the JGI using the Pacific Biosciences (PacBio) technology. A PacBio SMRTbell™ library was constructed and sequenced on the PacBio RS platform, which generated 403,200 filtered subreads totaling 1.1 Gbp. All general aspects of library construction and sequencing performed at the JGI can be found on the JGI website [http://jgi.doe.gov/]. The raw reads were assembled using HGAP (version: 2.0.12.0.1) [33]. The final draft assembly contained 171 contigs in 171 scaffolds, totalling 8.5 Mbp in size. The input read coverage was 181.5x.

### Genome annotation

Genes were identified using Prodigal [34] as part of the DOE-JGI genome annotation pipeline [35, 36]. The predicted CDSs were translated and used to search the National Center for Biotechnology Information nonredundant database, UniProt, TIGRFam, Pfam, KEGG, COG, and InterPro databases. The tRNAScanSE tool [37] was used to find tRNA genes, whereas ribosomal RNA genes were found by searches against models of the ribosomal RNA genes built from SILVA [38]. Other non–coding RNAs such as the RNA components of the protein secretion complex and the RNase P were identified by searching the genome for the corresponding Rfam profiles using INFERNAL [39]. Additional gene prediction analysis and manual functional annotation was performed within the Integrated Microbial Genomes platform [40] developed by the Joint Genome Institute, Walnut Creek, CA, USA [41].

## Genome properties

The genome is 8,458,965 nucleotides with 61.32 % GC content (Table 3) and comprised of 171 scaffolds of 171 contigs. From a total of 8,483 genes, 8,344 were protein encoding and 139 RNA only encoding genes. The majority of protein-coding genes (76.34 %) were assigned a putative function whilst the remaining genes were annotated as hypothetical. The distribution of genes into COGs functional categories is presented in Table 4.

**Table 3:** Genome statistics for *Ensifer* sp. PC2

Attribute	Value	% of Total
Genome size (bp)	8,458,965	100.00
DNA coding (bp)	7,124,539	84.22
DNA G + C (bp)	5,187,131	61.32
DNA scaffolds	171	100.00
Total genes	8483	100.00
Protein coding genes	8344	98.36
RNA genes	139	1.64
Pseudo genes	0	-
Genes in internal clusters	513	6.05
Genes with function prediction	6290	74.15
Genes assigned to COGs	5205	61.36
Genes assigned Pfam domains	6533	77.01
Genes with signal peptides	645	7.60
Genes with transmembrane helices	1733	20.43
CRISPR repeats	1	-

**Table 4:** Number of genes of sp. PC2 associated with general COG functional categories

Code	Value	%age of total (5,205)	Description
J	236	4.01	Translation, ribosomal structure and biogenesis
A	0	0.00	RNA processing and modification
K	514	8.74	Transcription
L	172	2.92	Replication, recombination and repair
B	2	0.03	Chromatin structure and dynamics
D	47	0.80	Cell cycle control, cell division, chromosome partitioning
Y	0	0.00	Nuclear structure
V	115	1.95	Defense mechanisms
T	271	4.61	Signal transduction mechanisms
M	331	5.63	Cell wall/membrane/envelope biogenesis
N	101	1.72	Cell motility
Z	0	0.00	Cytoskeleton
W	44	0.75	Extracellular structures
U	132	2.24	Intracellular trafficking, secretion, and vesicular transport
O	213	3.62	Posttranslational modification, protein turnover, chaperones
C	351	5.97	Energy production and conversion
G	548	9.31	Carbohydrate transport and metabolism
E	598	10.16	Amino acid transport and metabolism
F	116	1.97	Nucleotide transport and metabolism
H	277	4.71	Coenzyme transport and metabolism
I	227	3.86	Lipid transport and metabolism
P	309	5.25	Inorganic ion transport and metabolism
Q	171	2.91	Secondary metabolite biosynthesis, transport and catabolism
R	593	10.08	General function prediction only
S	395	6.71	Function unknown
X	120	2.04	Mobilome: prophages, transposons
-	3,278	38.64	Not in COGS

## Insights from the genome sequence

With a genome totaling 8.5 Mbp in size, *Ensifer* sp. PC2 is approximately 25 % larger than the average *Ensifer* genome in GenBank. Although PC2 shares 100 % 16S rRNA sequence identity and 99.17 Average Nucleotide Identity with *Ensifer* sp. TW10, also isolated from a Thar Desert woody legume, the genome of TW10 has a smaller size of 6.8 Mbp. PC2 contains over 1,000 genes that are not found in TW10, including two plasmid replication initiator proteins and a suite of genes (*vir*/*trb*) involved in conjugative transfer. From this it is assumed that the PC2 genome is multipartite and contains at least one conjugative plasmid. In PC2, 38.64 % of genes have not been assigned to a COG functional category, whereas in TW10, only 31.55 % have not been assigned to a COG functional category. Compared with TW10, PC2 has a much higher number of genes assigned to the mobilome category (54 and 120 genes, respectively) and to extracellular structures (29 and 44 genes, respectively).

## Conclusion

Based on the 16S rRNA gene alignment, *Ensifer* sp. PC2 is most closely related to *Ensifer* sp. TW10 and *Ensifer* sp. WSM1721, two strains isolated from perennial legumes growing in arid climates and alkaline soils in India and Australia, respectively [21, 42]. *Ensifer fredii* strains isolated from Chinese soybean were also superdominant in sampling sites with alkaline-saline soils [43], which suggests that the biogeographic distribution of several *Ensifer* spp. is linked to their adaptation to alkaline soils. Further, this suggests that the symbiotic associations formed by promiscuous legumes, such as *Prosopis*, are likely to vary depending on which rhizobial genera are best adapted to the edaphic conditions in which the host is growing.

The ability of PC2 to fix nitrogen with both *P. cineraria* (L.) Druce and the crop legumes *Vigna radiata* (L.) R.Wilczek and *V. unguiculata* (L.) Walp. makes it a valuable inoculant strain for use in arid, alkaline regions such as the Thar desert. Analysis of the PC2 sequenced genome and comparison with the genomes of sequenced *Ensifer* spp. and other rhizobia will provide insights into the molecular basis of the patterns seen in rhizobial biogeographic distributions and associations with plant hosts and into the molecular determinants of rhizobial tolerance to arid and alkaline environments.

## Additional files

Additional file 1: Table S1. Associated MIGS record for PC2 (DOCX 19 kb) Additional file 2: Table S2. Nodulation and N_2_ fixation properties of *Ensifer* sp. PC2 on selected legume hosts. (DOCX 17 kb)

## Abbreviations

ANI: Average nucleotide identity
GEBA-RNB: Genomic encyclopedia for bacteria and archaea-root nodule bacteria
IMG: Integrated microbial genomes
