# Supplementary material for: High-quality permanent draft genome sequence of Ensifer sp. PC2, isolated from a nitrogen-fixing root nodule of the legume tree (Khejri) native to the Thar Desert of India
Source: Stand Genomic Sci. 2016 Jun 23;11:43. doi: 10.1186/s40793-016-0157-7 (PMC4918122; doi:10.1186/s40793-016-0157-7)
Supplement: Additional file 1: Table S1. — Associated MIGS record for PC2 (DOCX 19 kb) [file 40793_2016_157_MOESM1_ESM.docx]

Additional file 1: Associated MIGS record

**Table S1.** Associated MIGS record for PC2

| **MIGS-ID** | **Field name** | **Description** | |
| --- | --- | --- | --- |
| **MIGS-1** | Submit to INSDC/Trace archives |  | |
| **1.1** | PID |  | |
| **1.2** | Trace Archive |  | |
| **MIGS-2** | MIGS CHECK LIST TYPE |  |  |
| **MIGS-3** | Project Name | GEBA - Root Nodulating Bacteria |  |
| **MIGS-4** | Geographic Location | Jodhpur, Indian Thar Desert |  |
| **4.1** | Latitude | 26.27061 |  |
| **4.2** | Longitude | 73.021177 |  |
| **4.3** | Depth |  |  |
| **4.4** | Altitude |  |  |
| **MIGS-5** | Time of Sample collection |  |  |
| **MIGS-6** | Habitat (EnvO) | Soil, root nodule, host |  |
| **6.1** | temperature | 28 |  |
| **6.2** | pH | 5-9.5 |  |
| **6.3** | salinity | 0.0 - 2.0% (w/v) |  |
| **6.4** | chlorophyll |  |  |
| **6.5** | conductivity |  |  |
|  |  |  |  |
| **6.6** | light intensity |  |  |
| **6.7** | dissolved organic carbon (DOC) |  |  |
| **6.8** | current |  |  |
| **6.9** | atmospheric data |  |  |
| **6.10** | density |  |  |
| **6.11** | alkalinity |  |  |
| **6.12** | dissolved oxygen |  |  |
| **6.13** | particulate organic carbon (POC) |  |  |
| **6.14** | phosphate |  |  |
| **6.15** | nitrate |  |  |
| **6.16** | sulfates |  |  |
| **6.17** | sulfides |  |  |
| **6.18** | primary production |  |  |
| **MIGS-7** | Subspecific genetic lineage | *Ensifer* sp. PC2 |  |
| **MIGS-9** | Number of replicons |  |  |
| **MIGS-10** | Extrachromosomal elements |  |  |
| **MIGS-11** | Estimated Size | 8,458,965 bp |  |
| **MIGS-12** | Reference for biomaterial or Genome report |  |  |
| **MIGS-13** | Source material identifiers | PC2, WSM4384 |  |
| **MIGS-14** | Known Pathogenicity | Non-pathogen |  |
|  |  |  |  |
| **MIGS-15** | Biotic Relationship | Symbiotic |  |
| **MIGS-16** | Specific Host | *Prosopis cineraria* |  |
| **MIGS-17** | Host specificity or range (taxid) | Mimosoideae and Papilionoideae spp. |  |
| **MIGS-18** | Health status of Host | Healthy; effective nitrogen fixation |  |
| **MIGS-19** | Trophic Level |  |  |
| **MIGS-22** | Relationship to Oxygen | Aerobe |  |
| **MIGS-23** | Isolation and Growth conditions | TY media, 28°C, aerobe |  |
| **MIGS-27** | Nucleic acid preparation | CTAB |  |
| **MIGS-28** | Library construction | Pacbio SMRTbell™ |  |
| **28.1** | Library size | 1.1 Gbp |  |
| **28.2** | Number of reads | 403,200 |  |
| **28.3** | vector |  |  |
| **MIGS-29** | Sequencing method | Pacific Biosciences RS platform |  |
| **MIGS-30** | Assembly |  |  |
| **30.1** | Assembly method | HGAP (version: 2.0.12.0.1) |  |
| **30.2** | estimated error rate |  |  |
| **30.3** | method of calculation |  |  |
| **MIGS-31** | Finishing strategy |  |  |
| **31.1** | Status | High-quality permanent draft |  |
| **31.2** | coverage | 181.5x |  |
| **31.3** | contigs | 171 |  |
| **MIGS-32** | Relevant SOPs |  |  |
| **MIGS-33** | Relevant e-resources |  |  |
