# Supplementary material for: High-quality permanent draft genome sequence of Ensifer sp. PC2, isolated from a nitrogen-fixing root nodule of the legume tree (Khejri) native to the Thar Desert of India
Source: Stand Genomic Sci. 2016 Jun 23;11:43. doi: 10.1186/s40793-016-0157-7 (PMC4918122; doi:10.1186/s40793-016-0157-7)
Supplement: Additional file 2: Table S2. — Nodulation and N2 fixation properties of Ensifer sp. PC2 on selected legume hosts. (DOCX 17 kb) [file 40793_2016_157_MOESM2_ESM.docx]

**Table S2**. Nodulation and N_2_ fixation properties of [*Ensifer*](http://dx.doi.org/10.1601/nm.1328) sp. PC2 on selected legume hosts.

| **Legume Species** | **Sub-family^a^** | **Wild/ Cultivar** | **Common Name** | **Habit** | **Nod^†^** | **Fix** |
| --- | --- | --- | --- | --- | --- | --- |
| [*Prosopis cineraria*](http://www.theplantlist.org/tpl1.1/record/ild-31185) (L.) Druce. | [Mimosoideae](http://www.ncbi.nlm.nih.gov/Taxonomy/Browser/wwwtax.cgi?lvl=0&id=3807) | Wild | Khejari | Tree | + | + |
| [*Leucaena leucocephala*](http://www.theplantlist.org/tpl1.1/record/ild-105) (Lam.) de Wit | [Mimosoideae](http://www.ncbi.nlm.nih.gov/Taxonomy/Browser/wwwtax.cgi?lvl=0&id=3807) | Wild | Subabul | Tree | + | + |
| [*Acacia saligna*](http://www.theplantlist.org/tpl1.1/record/tro-13024476) (Labill.) Wendl. | [Mimosoideae](http://www.ncbi.nlm.nih.gov/Taxonomy/Browser/wwwtax.cgi?lvl=0&id=3807) | Wild | Golden Wattle | Small Tree | + | + |
| [*Phaseolus vulgaris*](http://www.theplantlist.org/tpl1.1/record/ild-2934) L. | [Papilionoideae](http://www.ncbi.nlm.nih.gov/Taxonomy/Browser/wwwtax.cgi?lvl=0&id=3814) | Cultivar | Common bean | Annual Herb | - | - |
| [*Vigna radiata*](http://www.theplantlist.org/tpl1.1/record/ild-29556) (L.) Wilczek | [Papilionoideae](http://www.ncbi.nlm.nih.gov/Taxonomy/Browser/wwwtax.cgi?lvl=0&id=3814) | Cultivar | Mung bean | Annual Herb | + | + |
| [*Vigna unguiculata*](http://www.theplantlist.org/tpl1.1/record/ild-3589) (L.) Walp. | [Papilionoideae](http://www.ncbi.nlm.nih.gov/Taxonomy/Browser/wwwtax.cgi?lvl=0&id=3814) | Cultivar | Cowpea | Annual Herb | + | + |
| [*Macroptilium atropurpureum*](http://www.theplantlist.org/tpl1.1/record/ild-2784) (DC.) Urb. | [Papilionoideae](http://www.ncbi.nlm.nih.gov/Taxonomy/Browser/wwwtax.cgi?lvl=0&id=3814) | Cultivar | Siratro | Annual Herb | + | + |

^a^ Phylogeny according to [1]. **^†^** ‘+’ and ‘-’ denote presence or absence, respectively, of nodulation (Nod) or N_2_ fixation (Fix).
